# Factors influencing improved adherence and retention among men living with human immunodeficiency virus in sub-Saharan Africa: A scoping review

**DOI:** 10.4102/phcfm.v18i1.5309

**Published:** 2026-07-21

**Authors:** Lebogang G. Matonyane, Andrew Ross

**Affiliations:** 1 Department of Health Sciences, Faculty of Public Health and Family Medicine, University of KwaZulu-Natal, Durban, South Africa

**Keywords:** adherence, retention, men living with HIV, HIV care, sub-Saharan Africa

## Abstract

**Background:**

In sub-Saharan Africa (SSA), men are more likely than women to experience numerous treatment interruptions after receiving a human immunodeficiency virus (HIV) diagnosis and starting treatment. As this can lead to poor adherence and retention in care, there is a need to address it to achieve epidemic control.

**Aim:**

This scoping review aimed to synthesise current evidence on the factors influencing improved adherence and retention to care among men living with HIV in SSA.

**Method:**

A comprehensive literature search was performed across multiple electronic databases to identify studies published from 2019 to 2024. The authors independently analysed the titles and abstracts before exporting the articles to Endnote. Of the 550 studies identified in the peer-reviewed literature, 474 remained after removing 76 duplicates, with only nine being included after reading the full article.

**Results:**

The findings revealed three themes and six sub-themes related to improving adherence and retention: support for men (improve men’s HIV knowledge and peer support), digitalisation of antiretroviral therapy (ART) programme (appointment reminders) and improving access to ART services (community-centred services, differentiated model of care and flexible accessible services).

**Conclusion:**

Improving adherence and retention in care for men living with HIV requires a multifaceted approach that addresses the unique social, structural, family challenges and psychological barriers they face. Strategies should include targeted health education, stigma reduction and the integration of gender-sensitive services that resonate with men’s health-seeking behaviours.

**Contribution:**

This study summarised the strategies that can be put in place to improve adherence and retention in care among men living with HIV.

## Introduction

In the context of sub-Saharan Africa (SSA), men are more likely than women to experience numerous treatment intrusions after receiving a human immunodeficiency virus (HIV) diagnosis and starting treatment, leading to poor adherence and retention in care.^1,2,3,4^ Adherence to antiretroviral treatment (ART) is regarded as the willingness of people living with HIV (PLHIV) to be involved in their HIV treatment programme, and to start, manage and maintain a given therapeutic combination medication regimen to control their HIV replication and improve their immune function.^5^ The absence of treatment uptake and effective adherence among men has resulted in higher mortality rates than women living with HIV, with contributory factors including poor health-seeking behaviour, low treatment coverage, poor retention and a high rate of loss to follow-up (LTFU).^6,7,8^

Retention in care depends on successfully connecting, remaining and being actively engaged in HIV care, which is not an all-or-nothing process, as many patients cycle in and out of treatment.^9^ Patient retention in care is one of the important indicators of the success of ART programmes, with elevated levels mainly leading to improved adherence to ART, slow progression to acquired immunodeficiency syndrome (AIDS) and increased survival. Moreover, patients who are not in HIV care because of LFTU are likely to develop a high viral load, which is associated with an increased risk of infecting other people, including their partners.^10^

Suboptimal healthcare provider practices, including inadequate enhanced adherence counselling (EAC), abuse of patient confidentiality, poor offering of adherence counselling at each visit and drug stock-outs, specifically ART medication, have been demonstrated as significant health systems barriers to adherence among men living with HIV.^3,11,12,13^ Mainstreaming ART, as well as improving the hours of service availability in health care settings, can facilitate access and address ‘missed doses’ that are because of travel constraints and migration.^12^ Providing specific ‘morning’ and ‘evening’ ART centre hours may reduce work absenteeism and help manage time for men living with HIV, as they will be able to access services at convenience time.^14^ Long-distance truck drivers frequently experience difficulties with ART retention, as they are often on the road at the time of their appointments, and have limited access to healthcare facilities during their travels.^12^

Attrition to ART was particularly observed in younger men (specifically those < 35 years old), this high rate slowing regressing and degrading the gains made in HIV care over the years.^6^ Sustainable resources must focus on proven, scalable interventions to promote ART initiation and early retention among men in healthcare facilities.^15^ The goal of enhancing men’s participation in HIV services lies mainly at the social level, at community surroundings, workplace and places where they usually gather, such as sports grounds and taverns.^2^ However, the health care system must play its part by providing HIV services that enable easy access by men at times and locations that enable them to attend. Community-based outreach programmes and responsive male-friendly health services are also important strategies for improving the overall health of men and providing tailored healthcare services.^16^

Many ART programmes have been trying to identify and implement appropriate strategies to improve retention levels, specifically for men, which vary widely across health facilities and programmes within SSA,^16,4^ those clinics and programmes with higher levels being models for improvements.^10,4^ Benchmarking and peer learning are very important, and enable institutions not doing well with men’s retention programmes to learn strategies for improvements from those doing well as part of the quality improvement plan.^4^

To improve adherence and retention in care for men living with HIV, it is essential to synthesise the most recent literature from the past 5 years that considers various circumstances and methodologies. With the increase in the number of mobile phone users in SSA, there is a need for individuals and public health organisations to provide psychosocial support and ART adherence messages.^17^ In this regard, in 2017, over three-quarters of SSA’s population had a mobile phone.^17^ The literature synthesised from these studies may assist stakeholders to develop policies and implement strategies to ensure optimal adherence and retention in HIV care among men. The study, therefore, aimed to identify and outline the evidence available on factors influencing improved adherence and retention in care among men living with HIV in SSA.

## Methods

The scoping review is based on the methodological framework established by Arksey and O’Malley (2005), and is further enhanced by the work of Levac, Colquhoun and O’Brien.^18^ The framework consists of the following five stages: (1) identify the research question, (2) identify relevant studies, (3) select the study, (4) chart the data and (5) collate, summarise and report the results. This framework was chosen for its inclusivity, flexibility and iterative nature, as it does not impose strict methodological rules or require the evaluation of the quality of evidence.^19^ An additional parallel element is described regarding using a ‘consultation exercise’ to inform and validate the findings from the central scoping review, with consultation being considered an optional component of the study framework.^19^ The Preferred Reporting Items for Systematic Reviews and Meta-Analyses for Scoping Reviews (PRISMA-ScR) was utilised to detail how the articles identified during the search were sorted.

### Stage 1: Identify the research question

This scoping review began by defining the research question to ensure that the identified problem could be addressed, and appropriate objectives were developed to achieve the aim of identifying strategies to improve adherence and retention in care among men living with HIV in SSA. The research question for the review was: *What are the factors influencing improved adherence and retention in care among men living with HIV?*

### Stage 2: Identify relevant studies

A comprehensive literature search was performed across multiple electronic databases, including Education Resources Information Center (ERIC), PubMed, Index to Nursing and Allied Health Literature (CINAHL), MEDLINE (EBSCO) and Google Scholar, to identify relevant literature that had been published from 2019 to 2024. The timeframe was chosen to ensure the inclusion of the most recent research among this priority population. The search terms used to identify relevant articles included adherence, retention, men living with HIV, HIV care and sub-Saharan Africa (SSA), with all authors being involved in the literature search. The study utilised the Population, Concept and Context (PCC) framework to identify the review question’s main concepts and inform the search strategy (Table 1).

**TABLE 1:** Population, concept and context framework.

PCC elements	Description
Population	The study included men living with HIV who are already on the ART programme.
Concept	Therefore, the study aimed to identify and outline the evidence available on factors influencing improved adherence and retention in care among men living with HIV in SSA.
Context	Literature conducted at facility, community and workplace settings was considered for this review. The review considered cultural, geographic and racial contexts influencing men’s adherence and retention in HIV care.

ART, antiretroviral therapy; HIV, human immunodeficiency virus; SSA, sub-Saharan Africa; PCC, population, concept and context.

### Stage 3: Select the study

Articles were included based on specific inclusion and exclusion criteria.

#### Inclusion criteria

The inclusion criteria were English studies published from 2019 to 2024 in SSA countries, with the comprehensiveness and relevancy of the literature being prioritised. Although the primary focus was SSA, one high-quality randomised controlled trial conducted outside SSA was included because of its strong relevance to digital adherence strategies applicable to SSA contexts. The review included published scholarly articles that were peer-reviewed and available in full-text. They needed to focus on strategies to enhance adherence and retention in care among men 18 years and above who were living with HIV for at least 12 months and had tested positive using national testing algorithms. Those specifically dealing with retention and adherence for men living with HIV that had been implemented were included, with the possibility of scale-up to other parts of the world.

#### Exclusion criteria

Studies published before 2019 were excluded, as were those not focusing on either retention or adherence in men living with HIV, and aged younger than 18 years. In addition, newspapers, conference papers and other databases were excluded, as well as those reports with incomplete descriptions or insufficient details regarding whether or not the strategies worked, and their applicability at scale. Reviews and secondary studies are not included. The authors independently analysed the words in the title and abstract before exporting the full article to Endnote to eliminate duplicates. Table 2 summarises the databases used to search the literature, the search terms, and the inclusion and exclusion criteria for this review.

**TABLE 2:** Literature search, inclusion and exclusion criteria.

Literature research	Inclusion and exclusion criteria
Databases	ERIC PubMed CINAHL MEDLINE (EBSCO) Google Scholar
Search terms	Adherence Retention Men living with HIV HIV care SSA
Inclusion criteria	Conducted in the last 5 years, from 2019 to 2024, published in English Published scholarly articles, peer-reviewed and available in full-text Men aged ≥ 18 years living with HIV for at least 12 months since HIV diagnosis or since HIV treatment
Exclusion criteria	Focusing on men not living with HIV and those younger than 18 Newspapers, conference papers and other databases that were not discussed in this research Published prior to 2019 HIV negative men

HIV, human immunodeficiency virus; SSA, sub-Saharan Africa.

**FIGURE 1:**
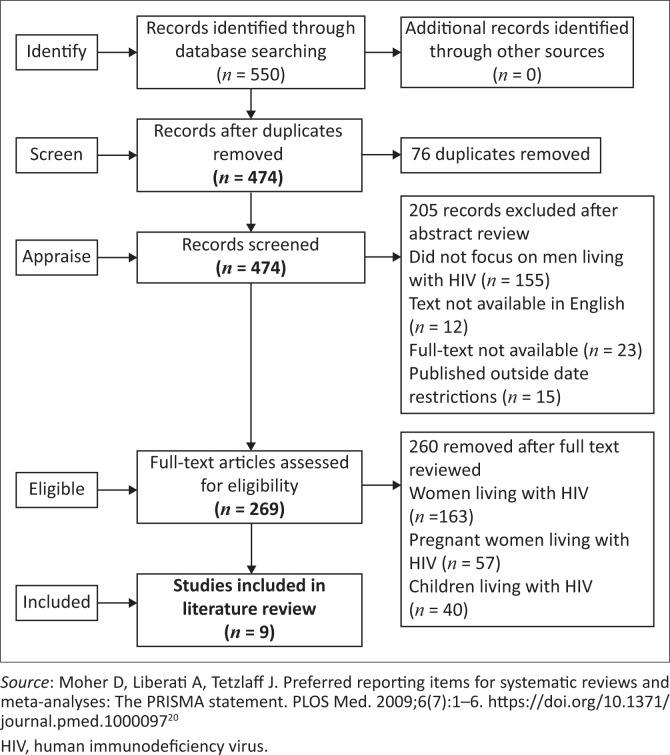
Flow chart of the search strategy.

The results from the various database searches, titles, abstract screening and full-text reviews were included. In total, 550 records were identified in peer-reviewed literature, of which 474 remained after removing duplicates (*n* = 76). The studies were screened by title and abstract, resulting in 269 full-text records being assessed for eligibility as per the inclusion and exclusion criteria (Figure 1). Of these, 260 were removed because of reporting on studies about children and women, which resulted in nine being included in the review.

### Stage 4: Chart the data

The studies extracted for this review included essential information, such as the authors, year of publication, country, study objectives, study design, population and sample characteristics and key findings related to the scoping review question, after a thorough full-text evaluation (Table 3).

**TABLE 3:** Literature included for review.

No	Author (s), year, and study topic	Purpose	Design	Sampling	Rigour or trustworthiness
1.	^22^Adeagbo O, Xulu Z, Gumede D, Naidoo K. 2024. Barriers and Strategies to Improve Men’s Uptake of HIV Care Services in Rural KwaZulu-Natal, South Africa: A Qualitative Approach	This study sought to *understand the key Barriers strategies or interventions* to effectively improve HIV service uptake among men and potential strategies to address these challenges	A qualitative study design was adopted	Purposive sampling was employed when recruiting participants, focusing specifically on those who did not utilise HIV services from the previous annual household survey	**Appraisal of quality grade is HQ = A** Purpose was clearly stated Strategy used as appropriate Findings were accurate and fair Objective methodology used Conclusion was highlighted.
2.	^40^Damulak PP, Ismail S, Abdul Manaf R, Mohd Said S, Agbaji O. 2021. Interventions to Improve Adherence to Antiretroviral Therapy (ART) in SSA: An Updated Systematic Review	Examines the effect of interventions in improving ART adherence in SSA, which bears the most considerable global burden of HIV infection	A systematic review using PRISMA guidelines	Systematic review	**Appraisal of quality grade is HQ = A** Purpose was clearly stated Strategy used as appropriate Findings were accurate and fair Objective methodology used Conclusion was highlighted.
3.	^28^Hlongwa M, Cornell M, Malone S, Pitsillides P, Little K, Hasen N. (2022). Uptake and Short-Term Retention in HIV Treatment Among Men in South Africa: The Coach Mpilo Pilot Project	Assess uptake and early retention in HIV care among men in the ‘Coach Mpilo’ peer support pilot project in South Africa	A pilot project conducted over 7 months (March 2020–September 2020).	Purposive sampling, men residing in the study areas were eligible for inclusion in the pilot	**Appraisal of quality grade is HQ = A** Purpose was clearly stated Strategy used as appropriate Findings were accurate and fair Objective methodology used Conclusion was highlighted.
4.	^30^Jiao K, Wang C, Liao M, Ma J, Kang D, Tang W, Tucker JD, Ma W. (2022). A differentiated digital intervention to improve antiretroviral therapy adherence among men who have sex with men living with HIV in China: A randomised controlled trial	To evaluate the effect of a differentiated digital intervention on ART adherence among men who have sex with men (MSM) living with HIV in China	A pragmatic two-armed parallel RCT, and inside each arm, there were three groups depending on the type of delivering the message and the type of information	Stratification was according to participants’ preferred digital strategy and block randomisation was triggered within each stratum	**Appraisal of quality grade is HQ = A** Purpose was clearly stated Strategy used as appropriate Findings were accurate and fair Objective methodology used Conclusion was highlighted.
5.	^25^Orlando S, Palla I, Ciccacci F, Triulzi I, Thole D, Sangare HM, Marazzi MC, Nielsen-Saines K, Turchetti G, Palombi L. 2021. Improving Treatment Adherence and Retention of HIV-Positive Women Through Behavioural Change Interventions Aimed at their Male Partners: Protocol for a Prospective, Controlled Before-and-After Study	To assess three interventions that *are aimed at* improving ART adherence and retention among HIV-positive *women*, through engagement with their male partners in four Malawian healthcare centres	A prospective controlled study suitable for evaluating promising interventions to improve male involvement and enhance maternal adherence to HIV PMTCT	Given the limited resources available for the study and practical difficulties related to the disadvantaged study context, the sample size calculations and criteria for assigning interventions were based on a pragmatic approach. Number of selected clusters limited to 4, one for each intervention plus one with no intervention as a control arm.	**Appraisal of quality grade is HQ = A** Purpose was clearly stated Strategy used as appropriate Findings were accurate and fair Objective methodology used Conclusion was highlighted.
6.	^23^Pugh LE, Roberts JS, Viswasam N, Hahn E, Ryan S, Turpin G, Lyons GE, Baral S, Hansoti B. 2022. A systematic review of interventions aimed at improving HIV adherence to care in low- and middle-income countries in sub-Saharan Africa	To comprehensively examine ART adherence and retention in care interventions in SSA and report on the implementation of interventions in real-world settings to inform future health investments in HIV care	A systematic review of studies examining interventions along the HIV care cascade	Systematic review	**Appraisal of quality grade is HQ = A** Purpose was clearly stated Strategy used as appropriate Findings were accurate and fair Objective methodology used Conclusion was highlighted.
7.	^27^Thurman TR, Luckett B, Zani B, Nice J, Taylor TM. (2024). Can Support Groups Improve Treatment Adherence and Reduce Sexual Risk Behaviour among Young people living with HIV? Results from a Cohort Study in South Africa	To investigate the effects of a structured support group intervention on treatment-related outcomes and sexual behaviour of AYLHIV in South Africa	A pragmatic, one-arm, pre-and post-intervention study conducted in urban communities in KwaZulu-Natal and Gauteng provinces of South Africa: Areas with the highest number and prevalence of adolescents living with HIV in the country	Using purposive sampling, participants recruited by community-based organisations serving to participate in Vhutshilo 3	**Appraisal of quality grade is HQ = A** Purpose was clearly stated Strategy used as appropriate Findings were accurate and fair Objective methodology used Conclusion was highlighted.
8.	^13^Ukoaka BM, Ugwuanyi EA, Ukueku KO, Ajah KU, Udam NG, Daniel FM, Wali TA, Gbuchie MA. 2024. Digital tools for improving antiretroviral adherence among people living with HIV in Africa	This comprehensive review synthesises evidence from existing research, identifies effective interventions and highlights gaps in knowledge	Literature review through online search across multiple electronic databases	Literature review	**Appraisal of quality grade is HQ = A** Purpose was clearly stated Strategy used as appropriate Findings were accurate and fair Objective methodology used Conclusion was highlighted.
9.	^21^WHO. 2023. Men and HIV: Evidence-based approaches and interventions A framework for person-cantered health services	This framework summarises barriers to health services experienced by men and strategies to address barriers and improve health service outcomes for men across three core pillars of person-cantered care: Access to care, quality services and supportive services	Clinical guideline	Clinical guideline	**Appraisal of quality grade is HQ = A** Purpose was clearly stated Strategy used as appropriate Findings were accurate and fair Objective methodology used Conclusion was highlighted.

Note: Please see the full reference list of the article Matonyane LG, Ross A. Factors influencing improved adherence and retention among men living with human immunodeficiency virus in sub-Saharan Africa: A scoping review. Afr J Prm Health Care Fam Med. 2026;18(1), a5309. https://doi.org/10.4102/phcfm.v18i1.5309, for more information

HIV, human immunodeficiency virus; SSA, sub-Saharan Africa; RCT, radomised controlled trial; PMTCT, prevention of mother-to-child transmission; AYLHIV, adolescent living with HIV; No, number.

### Stage 5: Collate, summarise and report results

The results of the nine studies were synthesised using a thematic narrative approach into themes and sub-themes based on the type of factors (strategies) influencing improved adherence and retention among men living with HIV.

## Review findings

### Results

Results line 3–5: The studies were of men living with HIV, published from 2021 to 2024. The countries of origin for these reviews were South Africa (SA) (*n* = 2), SSA (*n* = 2), Malawi (*n* = 1), Africa (*n* = 1) and China (*n* = 1). A policy document from the World Health Organization was also included. The included studies comprised three systematic or literature reviews, two qualitative studies, one pilot intervention study, one cohort study, one clinical guideline and one implementation framework, providing a broad range of methodological evidence relevant to the review question. In terms of the methods for the studies included for this review, they consisted of: three literature reviews, one qualitative and one quantitative study, three randomised controlled trials and one WHO policy document. The review identified peer-reviewed studies that reported one or more strategies for improving adherence and retention in HIV care among men living with HIV. The three themes identified were: support for men, digitalisation of ART programme and improving access to ART services, with a total of six sub-themes and 11 categories (Table 4).

**TABLE 4:** Themes, sub-themes and categories.

Themes	Sub-themes	Categories
1. Provide support for men	1.1. Improve men’s HIV knowledge	1.1.1. Tailored health education with counselling 1.1.2. Use of appropriate tools for communication
	1.2. Provide peer support	1.2.1. Psychosocial and emotional support 1.2.2. Adherence clubs 1.2.3. Peer-led support
2. Digitalise the ART programme	2.1. Appointment reminders	2.1.1. Short message services (SMS) or telephone monitoring system
3. Improve access to ART services	3.1. Community-centred services	3.1.1. Community support groups 3.1.2. Mobile health services
	3.2. Differentiated model of care	3.2.1. Multi-month dispensing of ART medication 3.2.2. Fast-tracking of services
	3.3. Flexible, accessible health services	3.3.1. Staggered hours of services

HIV, human immunodeficiency virus; ART, antiretroviral therapy.

### Theme 1: Provide support for men

It is very important for men living with HIV to receive ongoing support that is targeted at addressing their barriers to care health care facilities, as reports indicate that the health care system services available for men are often time bound and implemented with only a few male clients being reached.^3,21^ Public health services have historically not been developed with men in mind, often leaving them on the side-lines of key HIV and related health services necessary to improve morbidity and decrease mortality.^2^ Men also need responsive health services that align with person-centred care that re-orients them to put people and communities at the centre of service delivery strategies.^21^ This theme consisted of two sub-themes: to improve men’s HIV knowledge and to provide peer support.

#### Sub-theme 1.1: Improve men’s human immunodeficiency virus knowledge

Five studies (5, 6, 8, 9 and 6 in Table 3) referred to improving men’s HIV knowledge as an important strategy for enhancing adherence and retention in HIV care, these having been conducted in various places, thereby increasing the credibility of the findings. The studies reported that despite HIV having been a public health project for the last 30 years, many misconceptions and myths surrounding the condition affect people’s perceptions and actions, due in part to their having insufficient information. Ensuring that people are knowledgeable about HIV and how it could affect them needs to be consistent across all platforms, this being raised by the five studies.

Knowledge improvement was addressed through two categories: tailored health education counselling and the use of appropriate tools for communication. To achieve zero new infections and zero AIDS-related deaths of men in South Africa, efforts should be directed to developing tailored emotive educational and community-based strategies that address identified challenges to access to care to improve men’s utilisation of HIV care services.^22,3^

**Category 1.1.1: Tailored health education with counselling:** Ensuring that patients are educated does not appear sufficient to affect adherence levels independently.^23^ Men may have poor knowledge and motivation regarding HIV and related conditions, limited services available to prevent or treat conditions, and insufficient information about how to navigate health facilities to access health services successfully.^21^ Comprehensive counselling tailored to men’s particular concerns (e.g. wage-earning, sexual partners, children, lifestyle) and needs may help to improve their HIV-related knowledge, and hence improve retention in HIV care.^21^ Inadequate HIV information also plays an important role in rural men recognising their eligibility for HIV testing, with many having limited knowledge about HIV, which may lead to poor uptake of HIV programmes.^22^ In SSA, health messaging about HIV has often been fear-based, with men depicted as the reason for HIV spreading within communities in the region. Interviews with men, specifically those from communities with a high burden of HIV, show that they associate HIV with sickness and death, and regard a positive diagnosis as the end of their life as they know it.^24^

**Category 1.1.2: Using appropriate tools for communication:** A prospective, controlled before-and-after study design was conducted in three phases (total duration: 24 months): pre-intervention, intervention and post-intervention analyses. The Malawian study reported that an important aspect is the use of educational tools focused on messages, thereby initiating a reflective discussion of stereotypes and false beliefs related to the idea of masculinity present in the local culture.^25^ Digital tools are most effective when implemented in specific contexts to improve their effectiveness, with culturally sensitive design, consideration of local infrastructure limitations, and addressing digital barriers being essential for ensuring user engagement and maximising impact.^13^

#### Sub-theme 1.2: Peer support

Five studies (1, 3, 6, 7 and 9 in Table 3) reported on referred peer support as a key strategy for improving adherence and retention in HIV care among men living with HIV, this being addressed through psychosocial and emotional support, adherence clubs and peer-led support. Men have diverse experiences and identities, with specific but different health needs to those of women, making it important for them to be reached within their context, paying particular attention to vulnerable groups, including those members of key populations.^8,21^ Men may lack social connection and support with other men, specifically with respect to HIV and related conditions, with peer support being essential for many people living with HIV. Counselling and ongoing support from other men can provide meaningful relationships and connections for men.^21^ Three categories were identified in this sub-theme, these being, psychological and emotional support, adherence clubs and peer-led support.

**Category 1.2.1: Psychosocial and emotional support:** The use of peer role models should also be encouraged to educate men about HIV care. Moreover, friendly, confidential, community-based (e.g. mobile clinics) HIV care services for men could reduce stigma and encourage them to utilise the HIV services.^22^ Support from peers can provide the psychological and emotional support necessary to navigate the challenges of a stigma-related diagnosis of sexually transmitted infections (STIs), HIV and tuberculosis (TB), such as internal and external stigma. However, while there are numerous peer counselling and mentorship programmes tailored to adolescents and mothers living with HIV, very few are available that are led by men for men.^21^

**Category 1.2.2: Adherence clubs:** Adherence clubs, community-based adherence support groups and peer support groups positively impacted treatment adherence, with community-based support groups, rather than those directed through a clinic, being better for maintaining treatment success in those already adhering to ART.^23^ Clinic-based groups with a trained counsellor or community health worker (CHW) are effective, and may be particularly important for at-risk groups or those not already adhering to ART.^23^ Adherence clubs are also good opportunities to provide more wrap-around services, such as education, and learn about opportunities for additional support, such as food insecurity. Intervention with support groups can combine various services, including education, pill counting and home visits.^23^

**Category 1.2.3: Peer-led support:** A peer-led support group pilot project was conducted from March 2020 to September 2020 in three districts in South Africa: Ehlanzeni, Gert Sibande (Mpumalanga province) and Ugu district (KwaZulu-Natal province) for the Coach Impilo project, and revealed preliminary evidence that a peer-led support model was acceptable. It assisted with retaining a high proportion of men in the early stages of ART, with those living with HIV being invited to receive one-on-one coaching from a peer supporter who was stable on treatment.^26^ Some men relate to and prefer male-to-male support when discussing their challenges, particularly when they are private matters or related to sexual partnerships, as the supporters may be able to relate better to the struggles that they face.^21^ Better health and school attendance may be attributable to a combination of the psychosocial peer support received and improved treatment adherence.^8,27^

### Theme 2: Digitalise the antiretroviral therapy programme

Digital technologies and tools have been very important in providing solutions to the barriers to ART adherence and retention, such as stigma, digital divide, privacy concerns and limited healthcare services access.^13^ The digitalisation of ART delivery modalities has recently registered significant improvements, promising a paradigm shift in optimising care given to PLHIV.^13^ There was only one sub-theme for this theme, that being appointment reminders.

#### Sub-theme 2.1: Appointment reminders

Three studies (6, 8 and 9 in Table 3) conducted in different settings included SMS or telephone monitoring systems as a key strategy to improving adherence and retention in HIV care among men living with HIV. Health systems must offer services that minimise rather than increase barriers to care for men, the evidence suggesting that when men seek health services for themselves and other family members, their needs are often not met.^2,3,21^ While phone-based applications, such as short message services (SMS), phone calls and reminders, have charted an efficient course to date, the future of ART adherence monitoring resonates with the applicability of digital tools.^13^ A study that compared the effect of SMS appointment reminders on care retention in rural versus urban settings found that text messages were only effective for urban participants. This was because of challenges experienced with connectivity and the availability of cell phones, which hindered the effectiveness of this intervention in rural areas.^23^ This was addressed through one category: SMS or telephone monitoring systems.

**Category 2.1.1: Short message services or telephone monitoring system:** A systematic review found that SMS reminders were low cost and effective, and significantly increased medication adherence among PLHIV.^2^ Most text studies sent weekly reminders, with some sending them as often as daily.^23^ These interventions excluded individuals who did not have consistent mobile phone access could not read or respond to texts.^23^ Unfortunately, cost and technical know-how have hindered the availability and use of these tools in Africa, even among populations most deserving of these interventions, such as men. While no ‘gold standard’ for efficient adherence monitoring that exists, combining two or more digital tools has been advocated to improve retention in care among PLHIV.^13^ Men need services that are easy to access, delivered with quality, positive experiences, are responsive to their unique needs and enable the sustained uptake of health services.^21^

### Theme 3: Improve access to antiretroviral therapy services

Frequent facility visits and limited provision of services can restrict men’s income generation opportunities and risk unwanted disclosure of their HIV status, highlighting the need to ensure that services are accessible to them at a convenient time and place.^21^ This theme consisted of three sub-themes: community-centred services, differentiated model of care and flexible, accessible health services. While most men attend health facilities, community-centred and flexible health services provide convenient access points to those who are unable to attend primary health care settings, and can help reduce costs and stigma concerns.^2,21,^

#### Sub-theme 3.1: Community-centred services

Five studies (1, 3, 5, 6 and 9 in Table 3) referred to community-centred services as a key strategy to improving adherence and retention in HIV care among men living with HIV, this being covered through the two categories of community support groups and mobile services.

**Category 3.1.1: Community support groups:** Long waiting times, travel time and the costs related to attending health facilities can conflict with work demands for men, specifically mobile and primary wage-earning men.^21^ Some community-based HIV studies have shown modest or significant improvement in men’s uptake of HIV care services, with community support groups being used more often than peer, family and friend support.^22,28^ Community support programmes were more often associated with positive adherence outcomes than other support programmes.^23^ The intervention, called ‘Male Champion’, aims to change the beliefs and attitudes of men, not intervening directly on individuals but upon entire communities by involving community leaders.^25^ Some successes have been reported in community-based efforts in terms of enabling hard to reach men to access HIV services.^26^

**Category 3.1.2: Mobile health services:** Friendly, confidential, community-based (e.g. mobile clinics) HIV care services for men can reduce stigma and encourage more men to utilise them, but without addressing these intersections simultaneously, a meaningful approach to the ‘missing men’ phenomenon will not be realised.^22^ Community-centred or mobile services may benefit sub-populations who have additional challenges to facility attendance (such as young men), with high service coverage being essential for epidemic control to improve health outcomes.^22^ This strategy may reach the highest proportion of men quickly, particularly for populations that do not frequently attend health facility, such as young men.^21^ However, as the strategy is often more costly than other delivery models, locations where men gather, such as football games and trading centres, may reach a larger number.^21^

#### Sub-theme 3.2: Differentiated model of care

Three studies (4, 6 and 9 in Table 3) included a differentiated model of care as a key strategy in improving adherence and retention in care among men living with HIV, the two categories that emerged being multi-month dispensing of ART medication and fast-tracking services. Differentiated models of care involve providing services to men in places other than the health care facility, as they may choose to receive their treatment either at a community centre or a local pharmacy, which can be offered for more than 3 months if patients are stable on ART.

**Category 3.2.1: Multi-month dispensing of antiretroviral therapy medication:** Flexible facility-based services for men that offer differentiated services to improve access to care and retention in HIV care, such as multi-month dispensing (MMD), can be beneficial. This entails providing three to 6 months’ medication supply that can be accessed either at the local health facility or an external pickup point, and includes rapid medication refills without requiring patients to see a clinician.^21,3^ Reduced facility visits to access services can make adherence more manageable and accessible to men. In some studies, 6 months of ART dispensing improves long-term retention among established male clients.^21^ Men who are employed in highly mobile and informal occupations such as mining sector, agriculture environment, military service and long-distance transport experience occupation-related mobility that disrupts continuity of HIV care and retention on ART.^29^ Evidence from studies on mobile men living with HIV highlight that frequent travel and time spent on the road are associated with frequent treatment interruptions and missed appointments from the healthcare facilities. Mobile livelihood strategies, including long nights away for income generation, create logistical barriers to ART adherence and retention in care.^29^ There is a need to provide differentiated service delivery models tailored to mobile workers, including MMD, decentralised ART access at common destinations were these men meet or transit hubs, and flexible pre-travel refill systems to support retention in care among these occupational groups who are largely men.^29^ Increasing prescription length for stable patients.^23^

**Category 3.2.2: Fast-tracking services:** Streamlining care includes fast-tracking appointments, providing medication refill and integrated care systems such as treating families together.^23^ Emergency refills, whereby clients can access ongoing treatment refills at any health facility, are desired by mobile men and regarded as essential to sustained retention in HIV care. However, there is little evidence on the impact of emergency refills, a longer follow-up time being necessary to understand the sustainment over time of men on ART.^30^ Improving coverage of HIV services for men is essential for population health and HIV epidemic control.^21^

#### Sub-theme 3.3: Flexible, accessible health services

Two studies (1 and 9 in Table 3) highlighted flexible, accessible health services as a key intervention in improving adherence and retention in HIV care among men living with HIV, this being addressed through one category: staggered hours of services.

**Category 3.3.1: Staggered hours of service:** Flexible and efficient services that accommodate the time limitations related to men’s work schedules were regarded as important to improving their uptake of HIV services.^21^ A study conducted among sampled men aged 21 years to 65 years in rural KwaZulu-Natal province, SA, found that long waiting times because of queues was a barrier to services. Providing services during the evening and at weekends may be essential for men with fixed work hours, and should be considered based on the local context of those being targeted.^22^ Implementing evening or weekend hours has shown mixed results across various health service domains and settings, emphasising the importance of context in designing after-hours services.^21^

## Discussion

The aim of this scoping review was to identify and summarise factors that can be implemented in SSA to improve adherence and retention in HIV care among men living with HIV in an effort to reduce their morbidity and decrease the mortality rate. The study, therefore, aimed to identify and outline the evidence available on factors influencing improved adherence and retention in care among men living with HIV in SSA. The themes identified correspond to broader domains of support: theme 1 represents social and psychosocial support, theme 2 represents technological or medical support and theme 3 represents structural and health-system support. The scoping review steps were adapted from Arksey and O’Malley, with relevant designs and methods applied through the review.^19^

Support for men, digitalisation of ART programme, and improving access to services were the common themes that emerged from nine articles. They provided evidence that men who engaged in care need ongoing support within the healthcare system to avoid future treatment stoppages, leading to poor adherence and retention in care. The findings of this review can also be relevant in other parts of the world, where retention and adherence to HIV care also appear to be a challenge.

### Theme 1: Support for men

These findings corroborate the results of other studies, which documented that tailored health services for men living with HIV are essential to achieve optimal adherence and retention in care.^31,32,33,34,35^ Improving men’s knowledge of HIV is important to reduce stigma and improve medication adherence, with information being communicated using platforms such as social media, newspapers and during facility visits through health education. In the context of SSA, a strategy called entertainment education (‘edutainment’), which is an innovative and engaging mass media communication strategy that uses an entertainment medium, such as radio or television, to share messages that seek to bring about social and behavioural change, has been shown to improve retention in ART services.^24^ Addressing stigma as part of providing healthcare services for men is essential to address discrimination myths, misconceptions about HIV and the side effects of ART.^35^

Implementing supportive services, such as patient navigators, counselling incorporated into HIV education and providing tailored services, can lead to an increase in retention in care for PLHIV.^33^ Family support and safe disclosure of HIV status to partners or spouses emerged as an underlying facilitator of adherence based on the findings from this review. Disclosure enables practical support such as medication reminders, emotional encouragement and reduced stigma within the household environment, strengthening long-term retention in HIV care. The SA National Department of Health (DoH) has developed tools for communicating HIV information in the country’s 11 official languages to ensure that messaging is conveyed across all ethnic groups. Displaying posters and brochures in waiting rooms, having medical providers present brief messages to patients and clinics staying in close contact with patients over time can improve adherence and retention in HIV care.^31^

Similarly, men living with HIV need ongoing support, be it at the facility or from community members, including peers, family members and adherence clubs, to ensure that they are adherent to medication, this being particularly important when the patient is newly initiated on ART. The implementation of peer support groups, as a strategy to increase retention in HIV care and adherence to ART is essential to achieving zero HIV infection.^2,35,36^

Other findings suggest that hospital and clinic environments can be welcoming for patients, including men, and encourage them to use their services. However, this requires reducing the long waiting times, addressing the negative attitude of healthcare workers towards patients and the shortage of essential drugs, and providing responsive services. Adherence to ART and retention in care can be facilitated by good interpersonal relationships between hospital or clinic staff and patients through efficient service delivery at the facilities.^34^

### Theme 2: Digitalise the antiretroviral therapy programme

The advancement of cell phone technology could be used to assist the health care system to provide services to meet the needs of patients, including appointment and other service-related reminders, which can be done either through measures such as SMS. These findings are similar to a study by Khang et al., which noted that technological interventions have also improved retention in care through measures such as patient portals, appointment reminders, telehealth and clinic-based smartphone applications.^33^ The use of visual and verbal messages that emphasise the importance of retention in care are easy, low cost interventions that can be implemented by clinic staff where there is adequate data coverage.^32,34^ Contrary to our study and according to The Joint United Nations Programme on HIV/AIDS (UNAIDS), mass media approaches as part of technological interventions that challenge gender norms and link men to services have been shown to increase demand for men’s services, and support for women’s HIV services, particularly in high-prevalence areas in SSA.^4,24^ A combination of technology-based strategies should be implemented to enhance their individual chances of success in improving the uptake of services by men.

### Theme 3: Improving access to antiretroviral therapy services

Many working men are unable to take time off to visit healthcare facilities to get their ART medication without negative consequences for their pay. This challenge could be addressed by providing additional drug pickup points either in the community or near the workplace at designated sites or from mobile clinics.^36^ Providing separate, unique male health clinics and extended service hours for working men and women has been tried in various countries in SSA.^4^ This improved the uptake of health services among men, especially when accompanied by community-based promotion programmes and supported by peer referrals.^24^ Improving access to ART services is essential to improve adherence and retention in care for men living with HIV. Investment is needed to promote linkage to initiating ART, and for differentiated approaches to counselling for youth and those with identified suboptimal adherence. In addition, evidence from within Africa is needed on cost-effective strategies to identify and support PLHIV who are at an increased risk of non-adherence across the treatment cascade.^32^ Reducing waiting times and improving patient’s confidentiality and privacy can increase men’s use of health services.^24^ In Eswatini, men’s uptake of sexual health services improved when health care providers were more sensitive to their privacy concerns and time restrictions, and offered efficient services.^24^

Some community-based HIV studies have shown modest or significant improvements in men’s uptake of HIV care services.^37,38^ Research has shown that men who are living with HIV can be role models to other men to reduce HIV transmission.^39^ Opportunities for additional intervention strategies include using community-based organisations as a setting for engaging HIV-positive persons about the importance of regular care, and involving patients’ significant others in retention in care interventions.^31^ There is evidence that suggests that differentiated ART delivery models are particularly beneficial for men working in mining, agriculture, construction and other daily wage occupations where clinic attendance conflicts with income generation methods.^29^ In the context of SSA, there have been challenges with sustainability and equity when implementing community-based modalities as a result of the relatively high cost of providing such services.^24^

## Implications and recommendations

### Limitations

Some limitations may have affected the study, including the strict inclusion criteria focusing only on recent SSA studies may have limited the number of eligible studies included in this review. Only studies conducted in SSA were included. This review did not include sources such as newspaper articles, theses and books, which might provide helpful insight into the topic under investigation. Including studies in languages other than English and not limiting the search to those published in the last 5 years may have identified other approaches.

### Recommendations

To improve adherence and retention in care for men living with HIV in countries with a high burden of HIV, it is important to implement gender-sensitive, community-based strategies that address the unique challenges men face when accessing health services in different setting.^3^ A number of recommendations emanate from this study, including integrating HIV services into male-dominated settings, such as workplaces or sports clubs, offering flexible clinic hours, and utilising peer support models led by men who have lived experience. Digital health tools, such as SMS reminders, can also enhance engagement and retention in care. These findings suggest that involving men in the design and delivery of their care programmes could foster a sense of ownership and relevance, hence increasing their likelihood of sustained participation. Strengthening interpersonal counselling, family engagement and peer-led psychosocial support should be prioritised alongside structural interventions to improve retention in care among men.

## Conclusion

Improving adherence and retention in care for men living with HIV requires a multifaceted approach that addresses the unique social, structural and psychological barriers that they face. Strategies should include targeted health education, stigma reduction and the integration of gender-sensitive services that resonate with men’s health-seeking behaviours. Strengthening patient-provider relationships, enhancing access through flexible clinic hours, mobile health technologies and peer support networks can also significantly improve engagement. Fostering a supportive and non-judgemental care environment, combined with consistent follow-up and individualised support is essential to ensure long-term adherence and retention in HIV care among men.
